# C‐reactive protein is a prognostic biomarker in pancreatic ductal adenocarcinoma patients

**DOI:** 10.1111/ajco.13993

**Published:** 2023-07-06

**Authors:** Vanessa F. Bonazzi, Lauren G. Aoude, Sandra Brosda, Julia J. Bradford, James M. Lonie, Kelly A. Loffler, Michael G. Gartside, Kalpana Patel, Pamela Mukhopadhyay, Colm Keane, Val Gebski, James G. Kench, David Goldstein, Nicola Waddell, Andrew P. Barbour, the Australasian Gastro‐Intestinal Trials Group (AGITG) GAP investigators

**Affiliations:** ^1^ Frazer Institute The University of Queensland Woolloongabba Queensland Australia; ^2^ College of Medicine and Public Health Flinders University Bedford Park South Australia Australia; ^3^ QIMR Berghofer Medical Research Institute Herston Queensland Australia; ^4^ Mater Research Institute‐UQ South Brisbane Queensland Australia; ^5^ NHMRC Clinical Trials Centre Camperdown New South Wales Australia; ^6^ Royal Prince Alfred Hospital Camperdown New South Wales Australia; ^7^ University of Sydney Central Clinical School Camperdown New South Wales Australia; ^8^ University of NSW Prince of Wales Clinical School Randwick New South Wales Australia; ^9^ Princess Alexandra Hospital Woolloongabba Queensland Australia

**Keywords:** biomarker, C‐reactive protein, pancreatic adenocarcinoma

## Abstract

**Aim:**

The 5‐year survival rate of pancreatic ductal adenocarcinoma (PDAC) is approximately 11% and has only improved marginally over the last three decades. For operable PDAC, resection and adjuvant FOLFIRINOX chemotherapy is standard of care. There is growing interest in perioperative regimens to improve outcomes. The non‐randomized Phase II study “Gemcitabine and Abraxane for resectable Pancreatic cancer” (GAP) demonstrated the feasibility of perioperative gemcitabine/abraxane. Long‐term survival in PDAC requires an effective immune response; hence, we undertook this translational study of the GAP trial cohort to identify immune‐oncology biomarkers for clinical use.

**Methods:**

We combined Nanostring nCounter technology with immunohistochemistry to investigate the correlation between gene expression and overall patient survival. Findings were investigated in samples from the International Cancer Genome Consortium (ICGC, *n* = 88) and the Australian Pancreatic Genome Initiative (APGI, *n* = 227).

**Results:**

We confirmed that human equilibrative nucleoside transporter 1 (hENT1) expression was not a prognostic marker in PDAC but patients with high levels of hENT1 were more likely to live longer than 24 months post‐surgery. Additionally, *CD274* (PD‐L1) and two novel biomarkers of survival, cathepsin W (*CTSW*) and C‐reactive protein (*CRP*), were identified in the GAP cohort (*n* = 19). *CRP* expression was confirmed in data from the ICGC. Although PD‐L1 and CTSW proteins were not significant across all three cohorts, results show that low *CRP* mRNA and protein expression are associated with longer overall survival in all three patient groups.

**Conclusion:**

PDAC patients with long survival have higher hENT1 expression levels. Furthermore, *CRP* expression is a biomarker of poor prognosis following perioperative chemotherapy and resection in PDAC patients and thus may be useful for identifying patients who could benefit from more aggressive adjuvant strategies.

## Graphical abstract

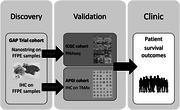

## 1 INTRODUCTION

Pancreatic ductal adenocarcinoma (PDAC) is the eighth most deadly cancer in Western societies.^1^, ^2^ PDAC prognosis has improved only marginally over the last three decades, with a 5‐year survival rate of approximately 11%.^3^ Standard of care for localized PDAC is surgical resection followed by adjuvant systemic therapy.^4^, ^5^ However, due to postoperative complications, recovery, and performance status, only 55%–65% of patients are able to receive adjuvant therapy.^6^ Neoadjuvant therapy is emerging as a viable strategy for PDAC, improving the proportion of patients with resectable or borderline resectable disease who are eligible to receive systemic and/or radiation therapy.^7^, ^8^

We recently published findings from the non‐randomized Phase II study “Gemcitabine and Abraxane for resectable Pancreatic cancer (GAP),” for patients with operable pancreatic adenocarcinoma.^9^ This trial assessed the feasibility and efficacy of neoadjuvant gemcitabine and nab‐paclitaxel, given for 8 weeks preoperatively and 16 weeks postoperatively. Resected tumor and peripheral blood samples were collected, along with clinical data. Results of the GAP trial determined that perioperative nab‐paclitaxel/gemcitabine therapy was safe and had the potential to improve surgical and long‐term outcomes. Furthermore, results of the Phase III APACT trial comparing adjuvant nab‐paclitaxel/gemcitabine with gemcitabine showed a significantly longer overall survival (OS) for nab‐paclitaxel/gemcitabine treated patients despite not reaching the primary objective comparing disease‐free survival.^10^ In the neoadjuvant setting, outcomes from randomized trials currently in progress are anticipated and will add to the understanding of the role of neoadjuvant therapy in PDAC.

PDAC is a heterogeneous disease,^11^ and novel therapeutic targets and biomarkers for PDAC are needed to improve patient outcomes following adjuvant and neoadjuvant therapies. One such biomarker is human equilibrative nucleoside transporter 1 (hENT1), which reflects response to gemcitabine in PDAC.^12^

Advances in immune cancer research have shown that the tumor immune microenvironment differentially impacts the activity of different classes of chemotherapies (antimetabolites, alkylating agents, microtubule‐targeting agents, taxanes, platinum compounds, and topoisomerase inhibitors) and improves the immune response.^13^ In PDAC, the tumor microenvironment plays a key role in the immunosuppressive response by decreasing stromal vascularization, altering immune cell infiltration and hypoxia, and attenuating drug delivery.^14^, ^15^, ^16^ Balachandran et al. have demonstrated tumor specificity with regard to the T‐cell clones that infiltrated the tumor.^17^ This was associated with unique neoantigens developed during primary tumor evolution of PDAC in patients with long‐term survival.^17^, ^18^ Understanding the immune landscape in PDAC will improve the development of novel therapeutic targets.

The aim of this study was to identify novel molecular markers of treatment efficacy and survival in patients with PDAC. Using patient samples from the GAP trial, we combined gene counts from Nanostring nCounter technology with immunohistochemistry (IHC) to investigate the relationship between gene expression and OS. A 770 pan‐cancer gene panel was used to classify tumors according to their immune response and identify differentially expressed genes as markers of survival.

## 2 METHODS

### 2.1 The GAP patient cohort

The study included patients from the Australasian Gastro‐Intestinal Trials Group (AGITG) non‐randomized Phase II GAP study. This trial combined perioperative chemotherapy with gemcitabine and nab‐paclitaxel. GAP was sponsored by the AGITG and coordinated by the NHMRC Clinical Trials Centre, University of Sydney, with recruitment from 2012 to 2014.^9^ It was registered with the Australian and New Zealand Clinical Trial Registry (ACTRN12611000848909). Informed, written consent was obtained from all participants in the GAP clinical trial (HREC/11/CIC/21). This sub‐study received ethics approval from the University of Queensland Ethics Committee (2019002388/LNR/2019/QMS/57218).

Forty‐two patients with radiologically defined resectable PDAC were enrolled on the trial and received two cycles of preoperative chemotherapy. Thirty‐six patients had surgery; however, six of these patients had unresectable tumors. Of the 30 patients who completed surgery, 12 were given postoperative chemotherapy. One patient had no pancreatic cancer after pathology review. For all patients, excess tumor tissue was collected at the time of surgery. Tissue was made into archival formalin‐fixed paraffin‐embedded (FFPE) samples, available for this sub‐study.

### 2.2 RNA extraction and Nanostring mRNA expression analysis

Hematoxylin and eosin slides from all surgical samples (*n* = 30) were reviewed by an experienced gastro‐intestinal anatomical pathologist (JGK). Samples from 19 patients had a tumor cellularity greater than 70% within the biopsy tissue and were selected for total RNA extraction. Between 5 and 10 sections (4‐μm) were cut from the FFPE blocks and used for extraction. Before transferring the tissues to extraction tubes, non‐tumor elements were removed via manual microdissection based on the hematoxylin and eosin slides. After the samples were deparaffinized using xylene, total RNA was extracted using RNeasy FFPE Kit (Qiagen) according to the manufacturer's protocol. RNA was quantitated using a Qubit 2.0 fluorometer (Invitrogen, Life Technologies), and their integrity was estimated using a 2100 Bioanalyzer (Agilent Technologies).

We performed mRNA expression analysis using the Nanostring nCounter Gene Expression Assay (NanoString Technologies) according to the manufacturer's protocol. The NanoString Human Pan‐Cancer Pathway Kit includes 770 genes that have been reported to be involved in 13 distinct cancer‐related pathways, including 40 housekeeping genes. The analysis and normalization were performed using nSolver software (NanoString Technologies) applying standard settings. Comparisons of gene expression were based on a fold change cutoff >1.2 and a *p*‐value <0.5.

### 2.3 ICGC RNA sequencing data

To confirm the immune response markers, we accessed RNA sequencing data from the International Cancer Genome Consortium (ICGC, Table S1). This included 88 treatment naive PDAC patients with operable disease.^11^ Data were previously published by Bailey et al.^19^ and are available in the EGA (accession number: EGAS00001000154). Data access was approved by the QIMR Berghofer Human Research Ethics Committee (HREC/P2905). Sequence reads were trimmed for adapter sequences using Cutadapt (version 1.11) and aligned using STAR (version 2.5.2)^20^ to the GRCh37 assembly. Quality control metrics were computed using RNA‐SeQC (version 1.1.8)^21^ and expression was estimated using RSEM (version 1.2.30).^22^ Library size was corrected for differences in RNA composition using trimmed mean of *M*‐values.

### 2.4 APGI tumor microarrays

Tumor microarray (TMA) samples, as well as related clinical data, were acquired from Australian Pancreatic Genome Initiative (APGI), HREC 2019002388/LNR/2019/QMS57218.^23^ A total of 227 PDAC primary tumors were represented on the TMA slides and included a subset of patients common to the ICGC cohort, *n* = 61 (26.8%, Table S1). In the total cohort, 18 out of 227 patients received neoadjuvant treatment, two patients had unknown treatment, and the remaining patients did not receive neoadjuvant therapy. Three tumor sections were included for each patient. Control tissues from several organs, including normal pancreas, were also included. The TMAs provided an independent patient cohort to validate findings from the GAP study.

### 2.5 IHC analysis

IHC was performed on the GAP trial FFPE samples and the APGI TMA samples to assess the protein expression of PD‐L1, cathepsin W (CTSW) and C‐reactive protein (CRP). After deparaffinization, hydration of sections, and antigen retrieval, sections were treated with peroxidase and then blocked. Slides were stained using the Discovery Ultra Ventana (Roche Diagnostics) with the following antibodies: hENT1,^12^ PD‐L1 (#13684; Cell Signaling Technology; 1:400); CTSW (ab191083; Abcam; 1:50) and CRP (ab32412; Abcam; 1:100). Human tonsils were stained as positive controls.

For the 19 GAP samples, staining was quantified by an anatomical pathologist (JGK) who estimated the percentage of positive cells. Staining intensity was described as: negative (0), weak but positive (1+), definite staining with intermediate intensity (2+), or strongly positive (3+). Results were scored by multiplying the percentage of positive cells by the intensity according to the following formula: *H*‐score = percentage positive cells × staining intensity score; maximum = 300. The *H*‐score was individually calculated for the tumor, the acini, and the infiltrated lymphocytes. For each marker, a combined *H*‐score is reported, range 0–900.

TMA slides were scanned (40×) on an Olympus VS120 Slide Scanner, and staining was quantified using the Visiopharm Image Analytical System (Version: 2017.2.4.3387). Positive marker staining was reported as a ratio of the marker staining compared to the assessed area which included the tumor cells, the infiltrated lymphocytes, and the stroma. For each patient, three individual tumor sections were scored and averaged.

### 2.6 Statistical methods

The GAP clinical trial provided follow‐up data to determine OS and progression‐free survival (PFS). OS was calculated from date of surgery until date of death from disease. PFS was the time from surgery until relapse. The primary endpoints of the study assessed hENT1 protein expression and OS, as well as immunotherapy genes and OS. The secondary outcomes of this study assessed the expression of these markers with PFS.

OS and PFS were analyzed using the Kaplan–Meier method (GraphPad Prism 7). Log‐rank Mantel–Cox tests were performed to determine whether there was a statistical difference between groups. The median marker expression was used to determine the optimal high/low cutoff. The difference was assessed using an unpaired *t*‐test (two‐tailed). For OS and PFS data, hazard ratios were determined using a Cox proportional hazards regression model (R Foundation for Statistical Computing).

## 3 RESULTS

### 3.1 GAP patient cohort

**FIGURE 1:**
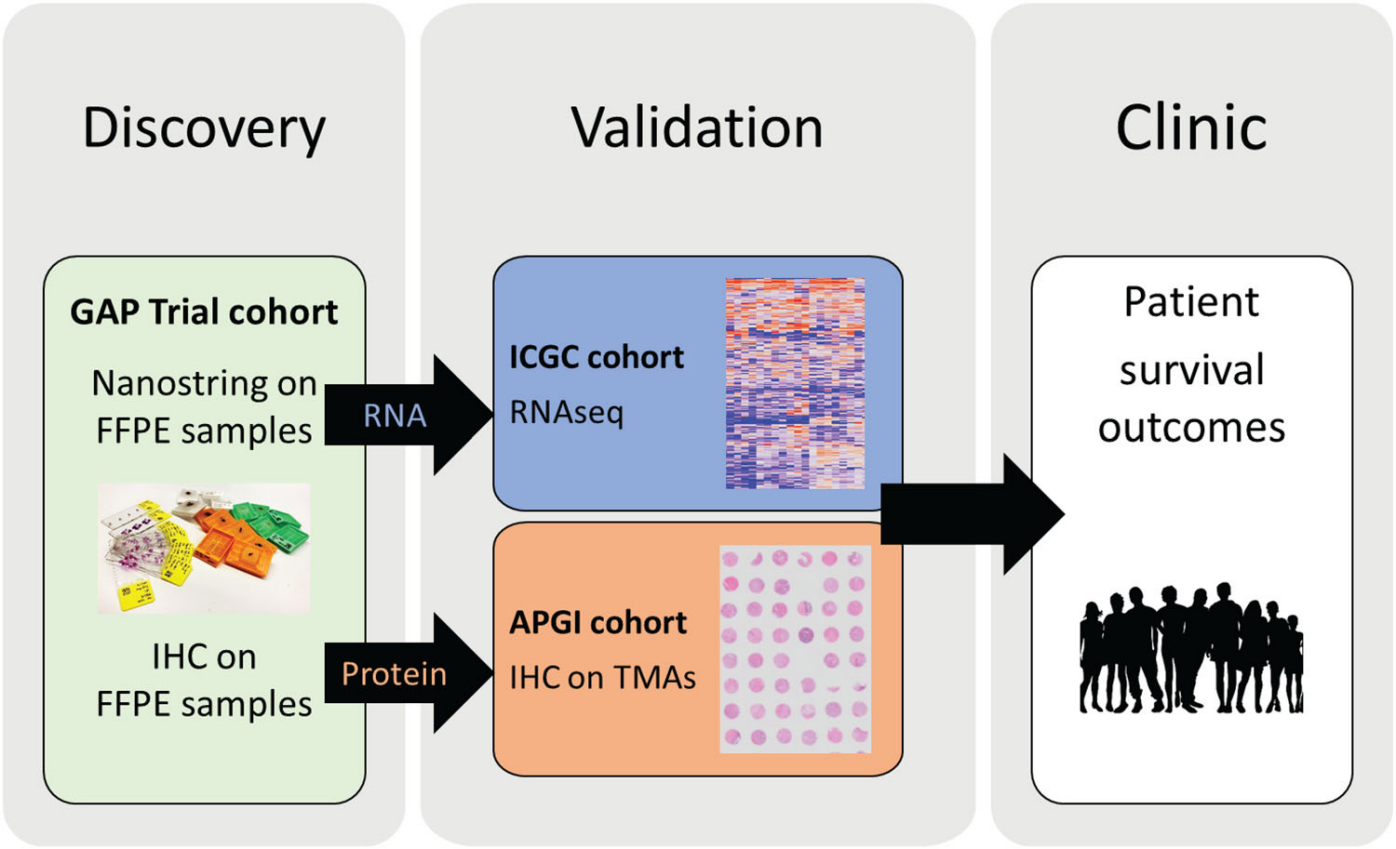
Study design. Biomarker discovery was performed in an archival tissue collection from patients enrolled in the Gemcitabine and Abraxane for resectable Pancreatic cancer (GAP) trial. RNA and protein expression were confirmed in two validation cohorts from the International Cancer Genome Consortium (ICGC) and Australian Pancreatic Genome Initiative (APGI). Biomarkers were correlated with patient survival outcomes to determine clinical utility.

We conducted a sub‐study of the GAP trial in which patients with resectable PDAC completed two cycles of preoperative chemotherapy (nab‐paclitaxel and gemcitabine) before surgery.^9^ Archival FFPE samples from a subset of 19 patients whose tumor was resected were used in this sub‐study (Figure 1). There were 13 females and 6 males, with ages ranging from 42 to 77 years (median 66 years). The median OS was 29.6 months (range, 5.0–50.3 months), and the median PFS was 11.66 months (range, 2.1–43.3 months). The median follow‐up time for survivors was 42.58 months (range, 38.51–45.11 months).

### 3.2 hENT1 protein expression, a marker of response to gemcitabine?

**FIGURE 2:**
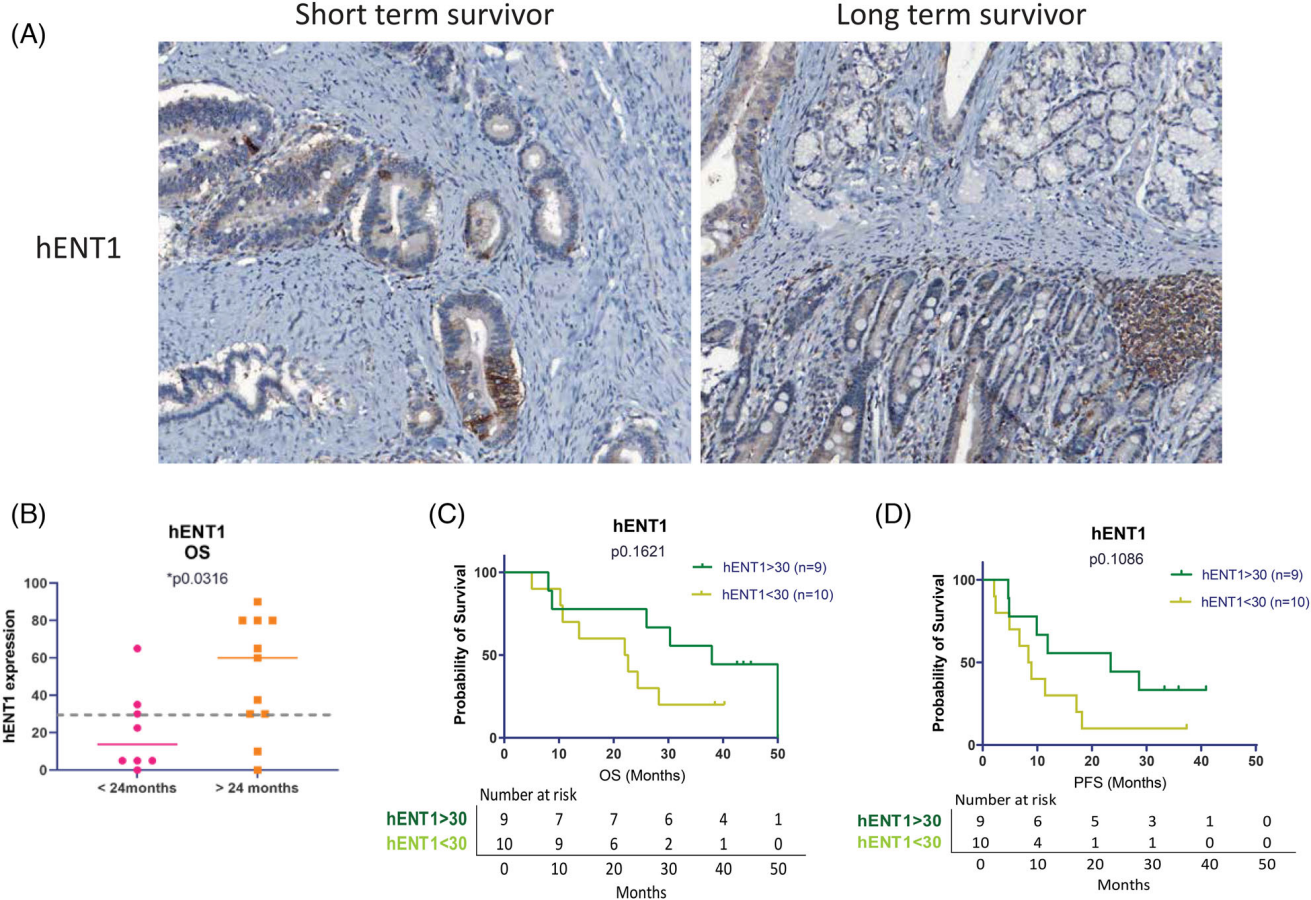
Human equilibrative nucleoside transporter 1 (hENT1), marker of response to Gemcitabine. (A) hENT1 staining in tumor from a patient with short overall survival (OS) compared to tumor from a patient with long OS. Original magnification 20×. (B) Dot plot comparing the spread of the immunohistochemistry (IHC) *H*‐scores calculated from staining in patients with short OS (<24 months) to long OS (>24 months) (*p* = 0.0316). The dotted line indicates median hENT1 expression. (C) Kaplan–Meier (log‐rank) survival analysis, curves split by median expression of hENT1 for OS (*p* = 0.1621) (D) Kaplan–Meier survival analysis, curves split by median expression of hENT1 for progression‐free survival (PFS) (*p* = 0.1086).

hENT1 expression has been previously shown to predict response to gemcitabine in pancreatic cancer.^12^ To assess hENT1 expression in the GAP cohort, we performed IHC analysis using previously published methods^12^ and obtained a median *H*‐score of 30, range 0–90 (Figure 2A). We found that patients with a short OS (<24 months) had weaker hENT1 IHC staining compared to patients with a long OS (>24 months) (*p* = 0.0316, log‐rank Mantel–Cox test) (Figure 2B). Using the overall median *H*‐score of 30 as the threshold, using univariate analysis, we did not see a significant correlation with hENT1 expression and overall (*p* = 0.1621, log‐rank) or PFS analyses (*p* = 0.1086, log‐rank, Figure 2C,D). We also confirmed no association between hENT1 expression and the age of diagnosis, as reported by Greenhalf et al.,^12^ and also no association with AJCC stage.

### 3.3 Identification of differentially expressed mRNAs

To identify specific immuno‐oncological biomarkers for PDAC in the GAP cohort, we used the Nanostring Pan‐Cancer pathways panel (Figure 1). Comparing gene expression in patients with short OS (<24 months) to patients with long OS (>24 months) revealed 323 differentially expressed genes (fold change >2, Figure S1A). Analysis of patients with a short PFS (<12 months) versus patients with a long PFS (>12 months) resulted in 362 differentially expressed genes (fold change >2, Figure S1B).

**FIGURE 3:**
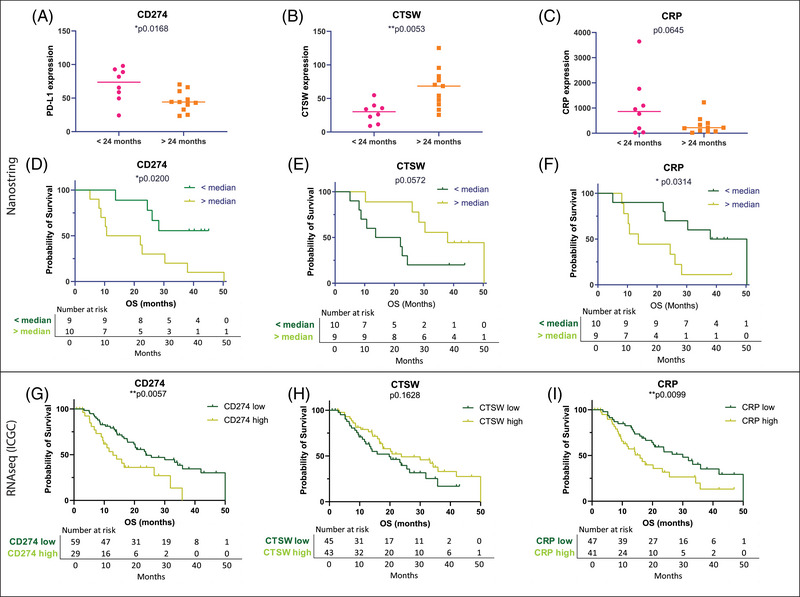
Differentially expressed mRNAs. The Nanostring panel determined differentially expressed genes between patients with short (<24 months) and long (>24 months) overall survival (OS). Two‐tailed unpaired *t*‐test analysis of (A) *CD274* (*p* = 0.0168). (B) Cathepsin W (*CTSW*) (*p* = 0.0053) (C) C‐reactive protein (*CRP*) (*p* = 0.0645). Kaplan–Meier (log‐rank) analysis of OS with groups split around the median expression of each marker. (D) *CD274* (median 49.5, *p = 0.0200). (E) *CTSW* (median 41.13, *p* = 0.0572). (F) *CRP* (median 220.26, *p = 0.0314). Survival analysis in an independent International Cancer Genome Consortium (ICGC) RNA sequencing dataset. Kaplan–Meier curves were stratified according to gene expression. (G) *PD‐L1* (**p = 0.0057). (H) *CTSW* (*p* = 0.1628, not significant). (I) *CRP* (**p = 0.0099).

Combining PFS and OS parameters, we found 22 common genes with fold change >1.2 and a *p*‐value <0.1 (Table 1). The volcano plots shown in Figure S1 indicate the significant genes common to both analyses which included two novel markers of survival: *CRP* (*p* = 0.061, unpaired *t*‐test; fold change = 4.28) and *CTSW* (*p* = 0.0013, unpaired *t*‐test; fold change = 2.54). Additionally, we included the *CD274* gene which encodes PD‐L1, an immune checkpoint expressed by activated T‐cells (*p* = 0.0569, unpaired *t*‐test). Two‐tailed unpaired *t*‐tests showed that patients with a long OS (>24 months) had lower *CD274* (*p* = 0.0168), lower *CRP* (*p* = 0.0645), and significantly higher *CTSW* expression (*p* = 0.0053, Figure 3A–C).

**TABLE 1:** Nanostring data, probe expression, fold change, and *p*‐values.

Probe	Expression OS > 24 months	Expression OS < 24 months	Fold change	*p* value	Expression PFS > 12 months	Expression PFS < 12 months	Fold change	*p* value
ADA	241.15	114.3	2.11	0.06	319.12	123.92	2.58	0.108
C6	418.82	301.66	1.39	0.57	656.79	256.91	2.56	0.091
CCL15	58.88	17.73	3.32	0.00	47.98	29.91	1.60	0.279
CCL25	41.39	12.08	3.42	0.08	36.07	19.78	1.82	0.522
CD274	42.63	60.06	−1.41	0.06	44.84	51.98	−1.16	0.382
CMA1	38.15	13.71	2.78	0.02	35.07	20.29	1.73	0.155
CRP	117.12	501.53	−4.28	0.06	84.22	376.44	−4.47	0.061
CTSW	58.95	23.18	2.54	0.00	54.74	33.07	1.66	0.071
CXCL5	202.32	685.02	−3.39	0.03	149.02	548.07	−3.68	0.050
CXCR2	37.26	71.02	−1.91	0.05	57.89	43.98	1.32	0.511
DMBT1	2772.83	916.21	3.03	0.06	1804.65	1714.83	1.05	0.942
FN1	4031.2	11,466.56	−2.84	0.01	3473.80	8848.67	−2.55	0.086
HLA–DQA1	25.85	196.38	−7.60	0.11	10.35	172.99	−16.71	0.014
HLA–DQB1	13.39	80.45	−6.01	0.06	9.64	53.90	−5.59	0.062
IL1B	87.81	97.44	−1.11	0.78	101.18	86.45	1.17	0.705
IL8	461.71	2074.35	−4.49	0.00	492.37	1209.56	−2.46	0.070
MME	139.08	64.06	2.17	0.14	219.52	63.08	3.48	0.083
S100A8	174	408.84	−2.35	0.01	211.16	273.80	−1.30	0.581
SAA1	242.69	769.08	−3.17	0.01	253.93	509.57	−2.01	0.177
TNF	28.8	32.05	−1.11	0.70	36.31	26.92	1.35	0.284
TREM1	111.59	307.06	−2.75	0.00	94.39	242.27	−2.57	0.006
USP9Y	9.82	24.91	−2.54	0.18	6.34	23.75	−3.75	0.060

Univariable survival analysis was performed to correlate mRNA expression of these markers with patient survival. For each marker, GAP patients were categorized into high/low groups using the median expression level as a cutoff. The median value for *CD274* was 49.5 (range 23.43–97.89). The median *CTSW* was 41.13 (range 9–125.21), and the median *CRP* value was 220.26 (range 18.65–3644.18). Patients with high *CD274* expression had significantly worse OS (*p* = 0.0200, log‐rank Mantel–Cox test, Figure 3D) and PFS (*p* = 0.0310) (data not shown). Patients with low expression of *CTSW* were found to have shorter OS (*p* = 0.0572, Figure 3E). Survival analyses showed that low levels of *CRP* were significantly associated with longer OS (*p* = 0.0314, log‐rank Mantel–Cox test, Figure 3F).

### 3.4 Validation in an independent RNA sequencing dataset from the ICGC

The results from the Nanostring analysis were evaluated in an independent dataset from the ICGC (*n* = 88) (Figure 1). The median OS of these patients was 15.85 months (range, 2.8–50.4 months), which is not statistically different from the GAP cohort (*p* = 0.3858, log‐rank, data not shown). In the ICGC dataset, poorer OS was associated with high *CD274* expression (*p* = 0.0057, Figure 3G) and high *CRP* expression (*p* = 0.0099, Figure 3I). *CTSW* expression showed no association with survival (*p* = 0.1628, Figure 3H). Results for *CD274* and *CRP* corroborated the observations from the GAP study.

### 3.5 IHC analysis to confirm biomarkers of survival in the GAP trial

We performed IHC to confirm the protein expression of these three gene biomarkers. From the 19 GAP patients with FFPE blocks, seven had been embedded in agar, which creates nonspecific background staining. Therefore, staining was performed on a subset of 12 samples (Figure S2). For each marker, the median *H*‐score was determined.

PD‐L1 is a transmembrane immune inhibitory receptor ligand that is expressed by T‐ and B‐cells, macrophages, and various types of tumor cells. For all 12 GAP patients, the acini and the tumor samples did not express PD‐L1. The tumor cells were girt by lymphocytes with weak staining profiles (PD‐L1 median *H*‐score = 0, range 0–4) (Figure S2).

The CTSW protein is a candidate tumor‐suppressor gene expressed by natural killer and cytotoxic T‐cells. In the GAP tumor samples, we observed nuclear staining in the acini, in the tumor cells, and also in the lymphocytes infiltrating the tumor (CTSW median *H*‐score = 460, range 90–810) (Figure S2).

The CRP staining revealed two types of samples. Six samples had acinar and tumor staining with few macrophages positive in the glands, whereas the other six samples had no staining in the tumor, but an increased number of positive macrophages infiltrated within the glands (CRP median *H*‐score = 350, range 10–600, Figure S2). As there were small numbers of FFPE blocks available for IHC analysis, no survival analysis was performed.

### 3.6 IHC analysis of the APGI cohort

**FIGURE 4:**
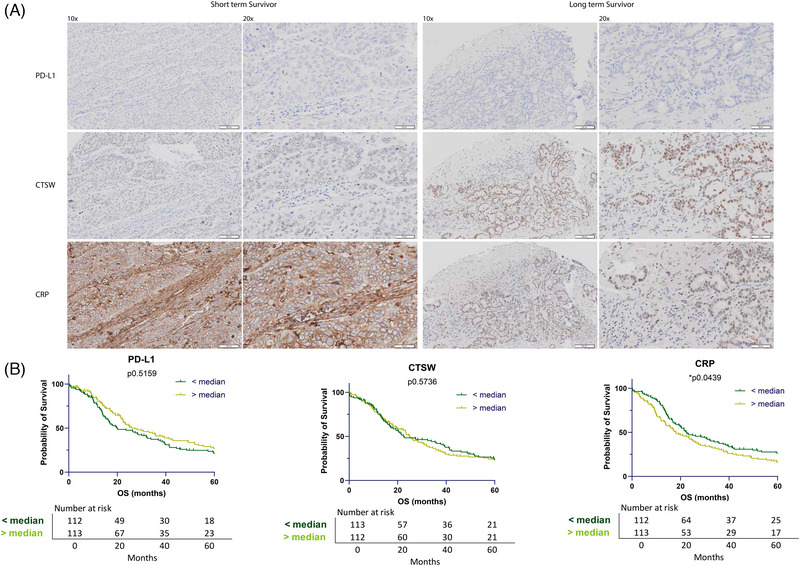
Immunohistochemistry (IHC) analysis of PD‐L1, cathepsin W (CTSW), and C‐reactive protein (CRP) in tumor microarray (TMA) samples from the Australian Pancreatic Genome Initiative (APGI). (A) Staining is shown in one patient with short overall survival (OS) (2.5 months) compared to one patient with long OS (115.5 months). Original magnification 10× and 20× as indicated, nuclei are counterstained with hematoxylin. (B) Kaplan–Meier (log‐rank) analysis of OS with groups split around the median expression of each marker. PD‐L1 (median 5.71, *p* = 0.5159), CTSW (median 3.98, *p* = 0.5736), CRP (median 35.72, *p = 0.0439).

In order to consolidate our findings and overcome the limitations of low sample numbers, we assessed TMAs from the APGI (Figure 1). Importantly, this validation cohort is treatment naïve. The median OS of these patients was 20.71 months (range 0–60). The median age of diagnosis was 67 years (range 34–88 years). The TMAs included tumor samples from 232 PDAC patients as well as control tissue. Survival analysis showed no statistical difference (log‐rank test) in OS when comparing the expression of PD‐L1 (median 5.71, range .16–798.26) or CTSW (median 3.98, range .07–52.78). However, we found that patients with low CRP expression (median 35.72, range 1.82–98.13) had longer OS (*p* = 0.0439, log‐rank), confirming the association from IHC and Nanostring analysis (Figure 4).

## 4 DISCUSSION

The study we present here is a follow‐up to the AGITG non‐randomized Phase II GAP study for patients with operable adenocarcinoma.^9^ We initially performed an analysis of hENT1 expression and confirmed previously published findings regarding its lack of prognostic impact.^12^ Our study could not be used to examine predictive value as all patients received gemcitabine. Nonetheless, we note that Greenhalf et al., in a much larger randomized study with fluorouracil and gemcitabine adjuvant therapy, investigated whether hENT1 expression was predictive of survival outcomes in PDAC patients that received adjuvant gemcitabine as a monotherapy but not in those who received fluorouracil. They concluded that although hENT1 has no prognostic significance, its expression levels could be used to direct choice of therapy. hENT1 is a promising predictive biomarker for PDAC for choice of neoadjuvant treatment. Those receiving preoperative gemcitabine/abraxane might be considered for an alternative adjuvant regimen such as FOLFIRINOX if their hENT1 expression is low. However, it is less useful as a prognostic tool. This highlights the need to develop more robust clinical markers that can better guide treatment escalation. We therefore sought to identify novel molecular biomarkers of survival in patients with PDAC from the GAP trial discovery cohort. Given the increasing evidence of a hostile immune microenvironment, using both RNA and protein expression, we investigated the correlation between immune‐oncology‐related biomarkers and OS in the GAP cohort, focusing on three biomarkers: PD‐L1, CTSW, and CRP.

Interaction of PD‐L1 with its receptor inhibits T‐cell activation and cytokine production. This interaction is essential to protect normal tissue and maintain homeostasis of the immune response during infection or inflammation.^24^, ^25^ In a tumor context, this PD‐L1/PD‐1 interaction changes the microenvironment and provides an immune escape for tumor cells through cytotoxic T‐cell inactivation.^24^ In many cancer types, the expression of PD‐L1 in tumor cells is considered a strong prognostic marker, and PD‐L1 is an emerging biomarker, especially in immunotherapy responsive tumors.^26^, ^27^, ^28^

There is a large variation in the techniques used to assess PD‐L1 protein level. The common variations across studies are in the scoring of tumor cells and the tumor‐infiltrating immune cells.^29^ In lung cancer cell lines, only a moderate correlation between *PD‐L1* mRNA expression and related protein has been reported to date (Pearson = 0.389).^30^ Unknown posttranscriptional mechanisms have been suggested to influence protein expression levels. PDAC patients present with a very low number of effector T‐cells, which creates an immune‐suppressive tumor microenvironment.^14^, ^31^ This is consistent with clinical trial outcomes showing a lack of efficacy of anti‐PD‐L1 treatments in PDAC.^32^, ^33^

We found that low *PD‐L1* expression was correlated with longer survival when assessed using RNA (Nanostring and RNAseq). However, when we analyzed PD‐L1 protein expression, we found no statistical correlation, which is in line with observations in other cancer types.^30^ As a biomarker, PD‐L1 remains problematic in PDAC as its protein expression is low in these tumors, which makes it difficult to assess in clinical pathology laboratories. As it may have a role in predicting response to immunotherapy in immunoresponsive tumors, more work is needed to clarify its utility in PDAC. The uncertainty of PD‐L1 as a reliable indicator of the degree of immunosuppression in the pancreatic microenvironment led us to examine alternative candidate biomarkers.

CTSW is a cysteine proteinase specifically expressed in natural killer cells and T‐cells, potentially regulating their T‐cell cytolytic activity. In other tumor types, *CTSW* has been reported as a candidate tumor suppressor gene, with expression positively correlated with patient survival and conferring a strong prognostic value.^34^, ^35^ Here, *CTSW* showed promising results in the Nanostring data, with high mRNA expression correlating with longer OS. However, during the validation process, in the ICGC and the APGI cohorts, *CTSW* mRNA and protein expression no longer correlated with survival. This highlights the importance of independent validation cohorts to identify specific biomarkers.

CRP is a protein produced in the liver during the acute phase response to tissue injury, infection, or other inflammatory stimuli.^36^, ^37^ In accordance with this study, other groups have shown that increased levels of CRP in the blood are associated with poor clinical outcome in advanced inoperable esophageal cancer^38^ and in pancreatic cancer.^39^, ^40^, ^41^ In pancreatic cancer, blood CRP levels were specially related to tumor size.^42^ Interestingly, in their study, Wang et al. concluded that NLR (the neutrophil‐to‐lymphocyte ratio) was a better prognostic biomarker than other inflammation‐related factors in patients with pancreatic cancer.^43^ Our study is the first to describe CRP expression in the tumor tissue of PDAC patients.

We examined the differences in treatment schedules between the GAP trial patients and the APGI cohort. Patients on the GAP trial received neoadjuvant chemotherapy, whereas the APGI patients received chemotherapy post‐surgery. As the validation cohort from the APGI was treatment naïve, CRP expression is independent of treatment type. This suggests that it could be a worthy biomarker to further develop in a clinical setting.^44^, ^45^, ^46^

Subsequent studies have continued to validate markers of a pro‐inflammatory microenvironment, such as NLR, as independently prognostic for outcome. This indicates that a therapeutic pathway of reprogramming the microenvironment may well enable more successful immunotherapy approaches to treatment of pancreatic cancer.^47^, ^48^, ^49^

Our study is limited by the availability of samples, which is a challenge for all research groups working on pancreatic cancer. There are very few studies in the neoadjuvant setting as it is still an emerging area of interest. The GAP trial had a small number of patient samples available (*n* = 19). Recent studies focusing on this topic have presented results from 12^50^ or 24^51^ patients. The largest PDAC study examining gene profiles contained 1200 patients but did not report relationships with survival outcomes.^52^ To overcome this hurdle, we have validated our findings in an independent dataset from the ICGC (*n* = 88) as well as a second validation cohort from the APGI (*n* = 232). This provided a robust method for validating our novel biomarkers and their association with patient survival.

In summary, we have suggestive evidence of the value of hENT1 protein expression as a potential marker in the neoadjuvant setting that indicates the need for expanded sample sizes. Further validation is also required to confirm CRP as a prognostic factor for survival following gemcitabine/abraxane treatment and resection. These data should be considered stratification factors in future clinical studies to determine the efficacy of new regimens in the increasing role of neoadjuvant therapy.

## AUTHOR CONTRIBUTIONS

Vanessa F. Bonazzi, Lauren G. Aoude, Michael G. Gartside, and Andrew P. Barbour contributed to the study design, methodology, and data analysis. Andrew P. Barbour and David Goldstein recruited the patients as part of the GAP trial. Val Gebski led the statistical analysis of the GAP trial. Michael G. Gartside, Kelly A. Loffler, Kalpana Patel, and Colm Keane performed laboratory experiments related to tissue samples. Sandra Brosda, Pamela Mukhopadhyay, and Nicola Waddell contributed to the acquisition and analysis of sequencing data. Vanessa F. Bonazzi, Julia J. Bradford, and James G. Kench performed IHC scoring and analysis. Vanessa F. Bonazzi, Lauren G. Aoude, and James M. Lonie wrote the manuscript. All authors edited, read, and approved the final manuscript.

## CONFLICT OF INTEREST STATEMENT

NW is a founder and Board member of genomiQa. NW is a member of the executive committee of the APGI. The other authors declare no potential conflicts of interest.

## ETHICS STATEMENT

GAP was sponsored by the AGITG and coordinated by the NHMRC Clinical Trials Centre, University of Sydney. It was registered with the Australian and New Zealand Clinical Trial Registry (ACTRN12611000848909). All participants provided written informed consent in the GAP clinical trial (HREC/11/CIC/21). This sub‐study received ethics approval from the University of Queensland Ethics Committee (2019002388/LNR/2019/QMS/57218). mRNA sequencing data access was approved by the QIMR Berghofer Human Research Ethics Committee (HREC/P2905).

## Supporting information

Table S1 Clinical information for the ICGC and APGI cohorts includes histo‐subtype, treatment, and survival.

Figure S1 Volcano plots depicting the p value of differentially expressed genes as a function of fold change between the indicated groups. A. OS > 24 months versus OS < 24 months. B. PFS > 12 months versus PFS < 12 months. Colored dots indicate CD274, CTSW, and CRP, principal markers investigated in this study.
Figure S2 IHC analysis of PD‐L1, CTSW, and CRP in short term (9.66 months) and long‐term (30.32 months) overall survivors from the GAP trial. IHC staining is shown in the acini and in the tumor. PD‐L1, CTSW, and CRP peroxidase staining show as brown, and nuclei are counterstained with hematoxylin. Original magnification 10× and 20× as indicated.

## Data Availability

The Nanostring data that support the findings of this study are available from the corresponding author upon reasonable request. mRNA sequence data included in this study are openly available in the European Genome‐phenome Archive, study ID EGAS00001000154, dataset ID EGAD00001003298.
