# Supplementary material for: C‐reactive protein is a prognostic biomarker in pancreatic ductal adenocarcinoma patients
Source: Asia Pac J Clin Oncol. 2023 Jul 6;21(1):77–86. doi: 10.1111/ajco.13993 (PMC11733851; doi:10.1111/ajco.13993)

Figure 1

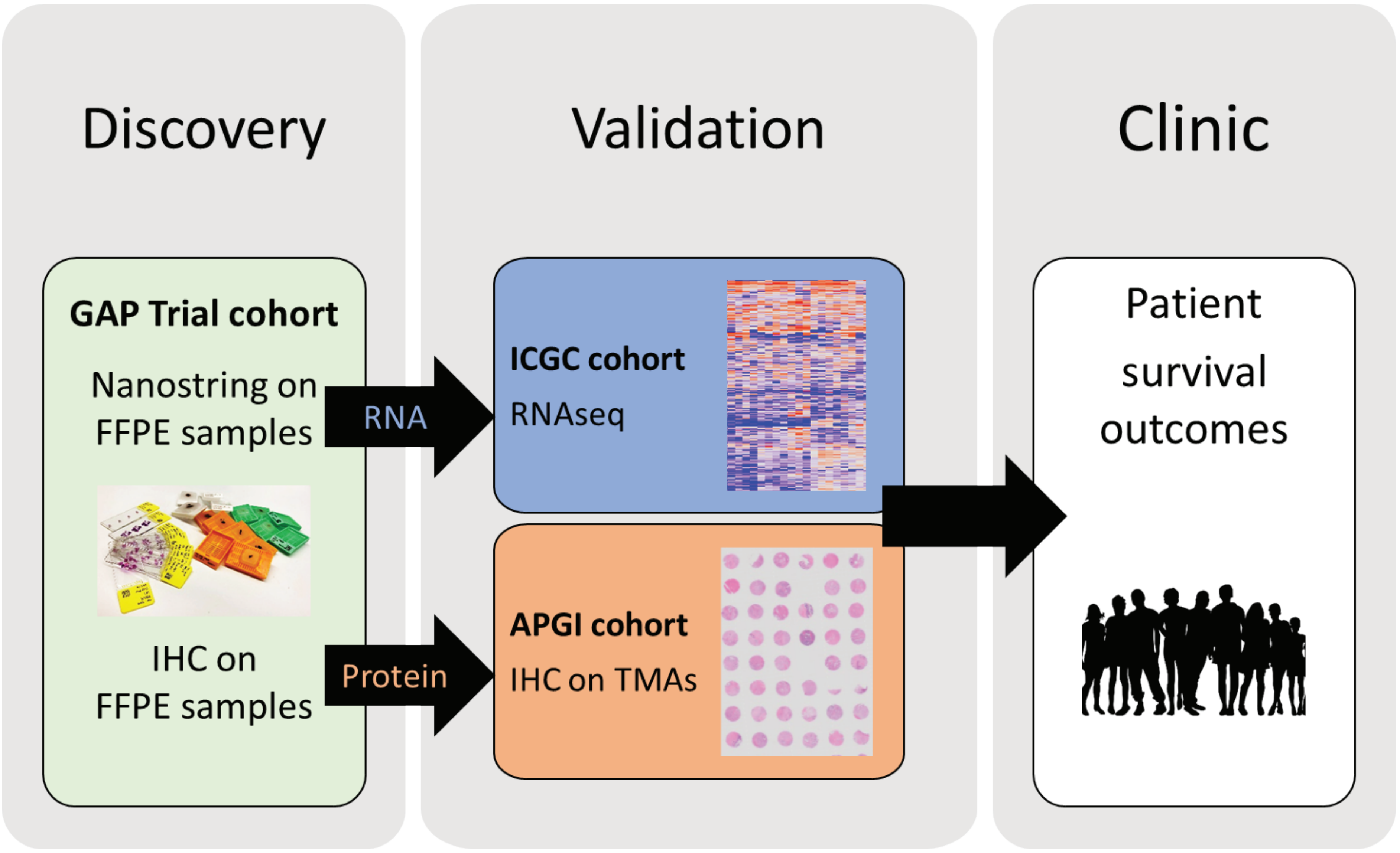
Discovery
Validation
Clinic
GAP Trial cohort
Nanostring on
FFPE samples
IHC on
FFPE samples
RNA
Protein
ICGC cohort
RNAseq
APGI cohort
IHC on TMAs
Patient
survival
outcomes

Figure 2

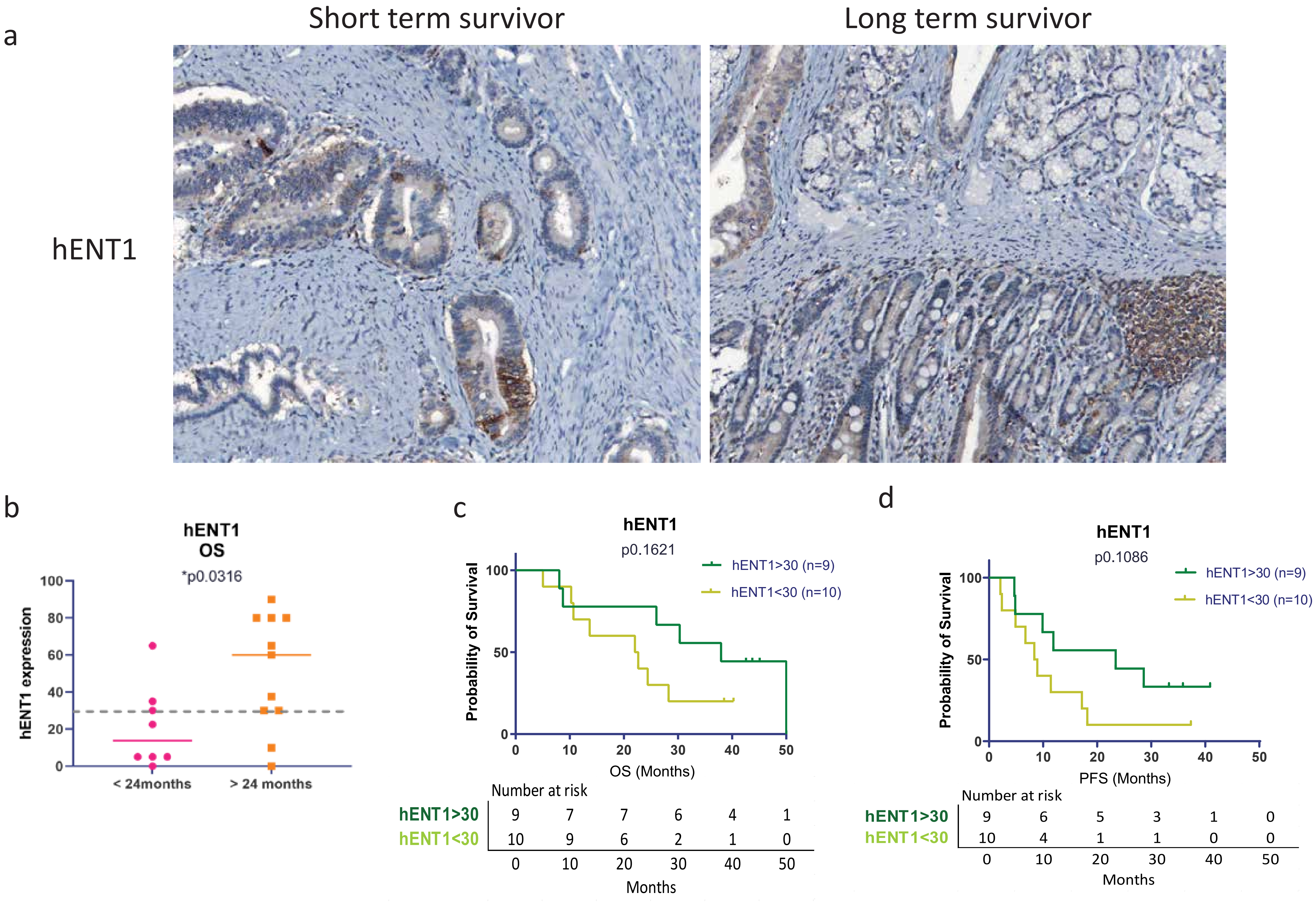

a
Short term survivor
Long term survivor
hENT1
b
hENT1
OS
*p0.0316
hENT1 expression
100
80
60
40
20
0
< 24months
> 24 months
c
hENT1
p0.1621
hENT1>30 (n=9)
hENT1<30 (n=10)
Probability of Survival
100
50
0
0 10 20 30 40 50
OS (Months)
Number at risk
hENT1>30 9 7 7 6 4 1
hENT1<30 10 9 6 2 1 0
0 10 20 30 40 50
Months
d
hENT1
p0.1086
hENT1>30 (n=9)
hENT1<30 (n=10)
Probability of Survival
100
50
0
0 10 20 30 40 50
PFS (Months)
Number at risk
hENT1>30 9 6 5 3 1 0
hENT1<30 10 4 1 1 0 0
0 10 20 30 40 50
Months

Figure 3

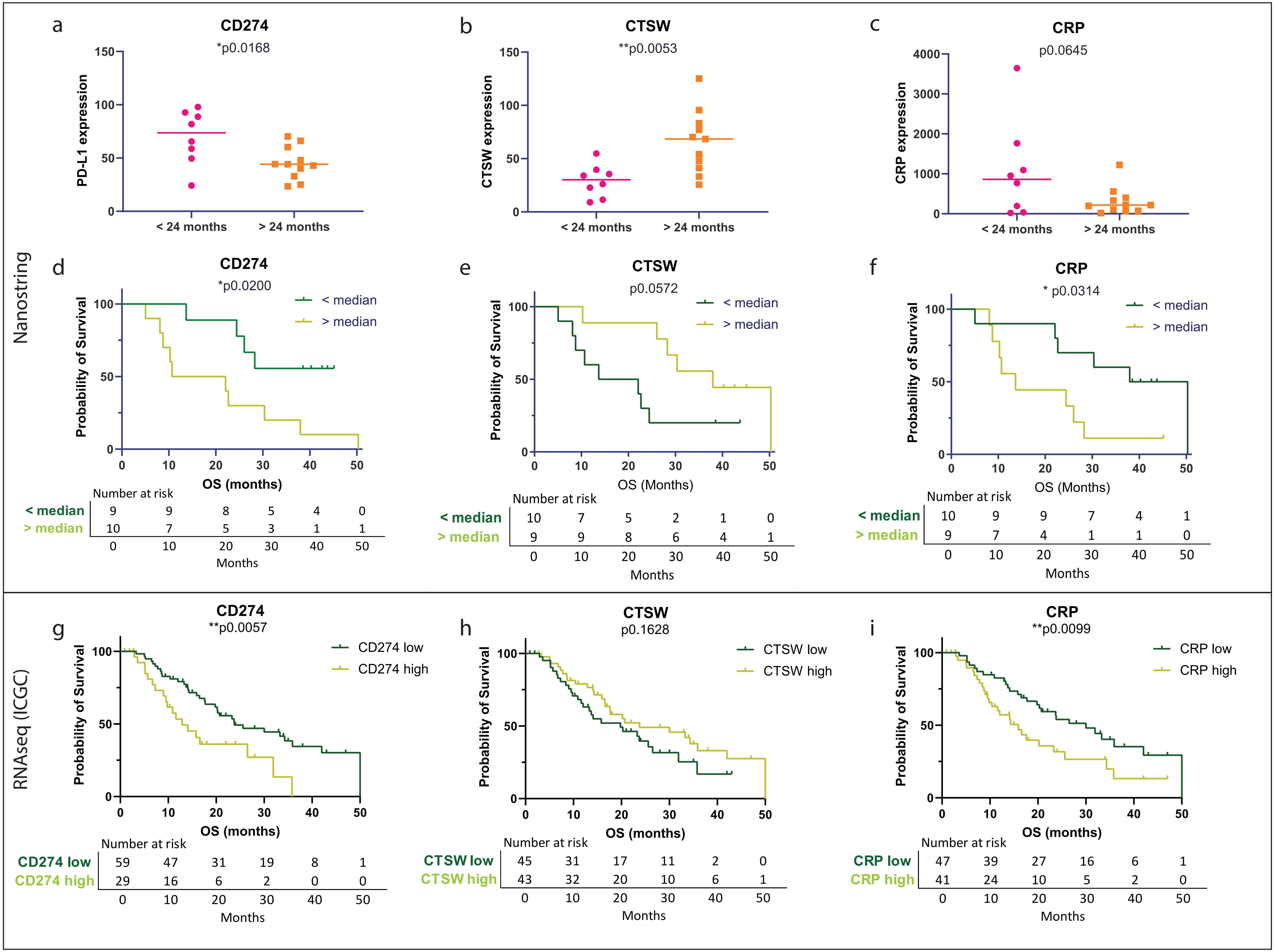

Nanostring
RNAseq (ICGC)
a
CD274
*p0.0168
PD-L1 expression
< 24 months
> 24 months
b
CTSW
**p0.0053
CTSW expression
c
CRP
p0.0645
CRP expression
d
CD274
*p0.0200
Probability of Survival
OS (months)
< median
> median
Number at risk
< median 9 9 8 5 4 0
> median 10 7 5 3 1 1
Months
e
CTSW
p0.0572
OS (Months)
< median 10 7 5 2 1 0
> median 9 9 8 6 4 1
f
CRP
* p0.0314
< median 10 9 9 7 4 1
> median 9 7 4 1 1 0
g
CD274
**p0.0057
CD274 low
CD274 high
CD274 low 59 47 31 19 8 1
CD274 high 29 16 6 2 0 0
h
CTSW
p0.1628
CTSW low
CTSW high
CTSW low 45 31 17 11 2 0
CTSW high 43 32 20 10 6 1
i
CRP
**p0.0099
CRP low
CRP high
CRP low 47 39 27 16 6 1
CRP high 41 24 10 5 2 0

Figure 4

A

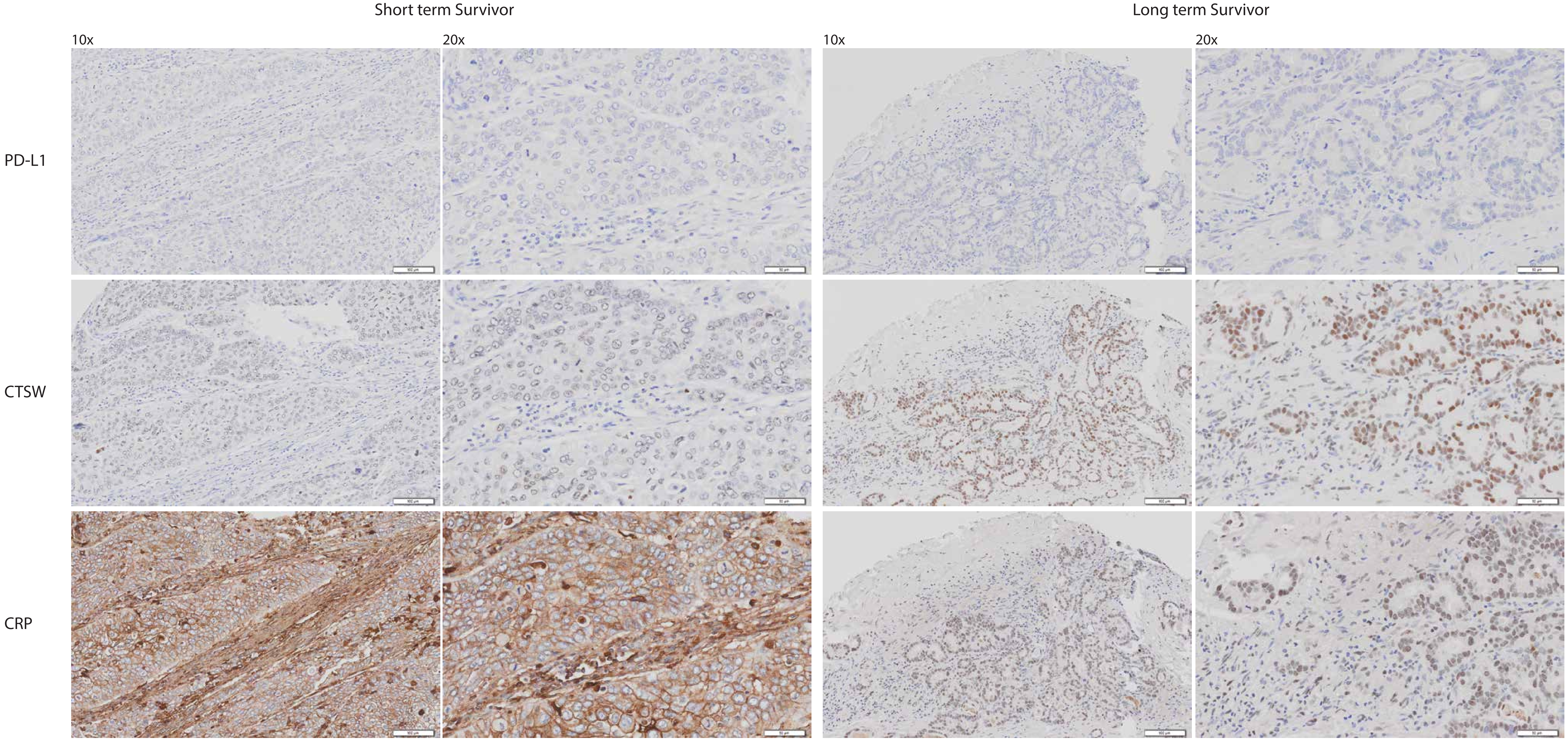

B

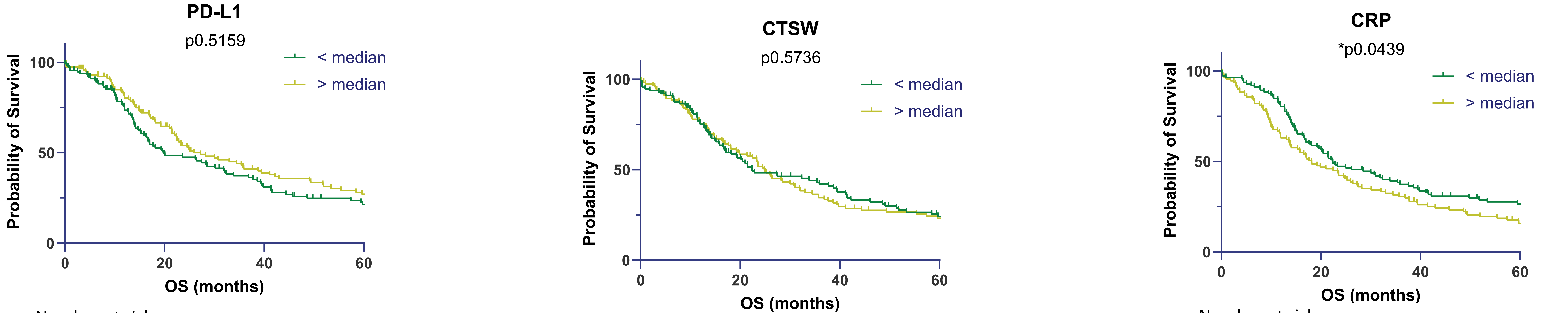

PD-L1 – Number at risk

	0	20	40	60
**< median**	112	49	30	18
**> median**	113	67	35	23

Months

CTSW – Number at risk

	0	20	40	60
**< median**	113	57	36	21
**> median**	112	60	30	21

Months

CRP – Number at risk

	0	20	40	60
**< median**	112	64	37	25
**> median**	113	53	29	17

Months

Supplementary Figure 1

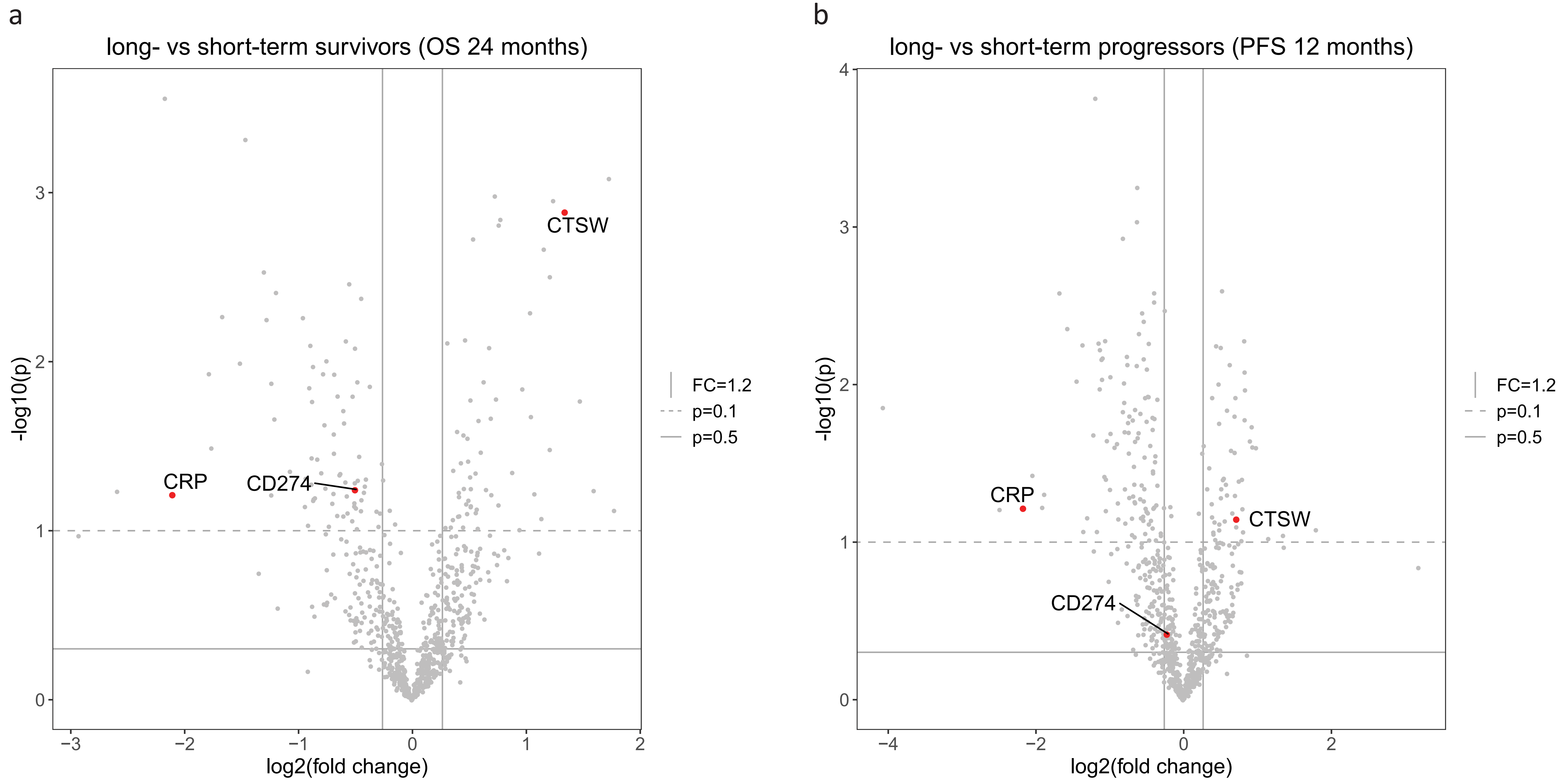

Supplementary Figure 2

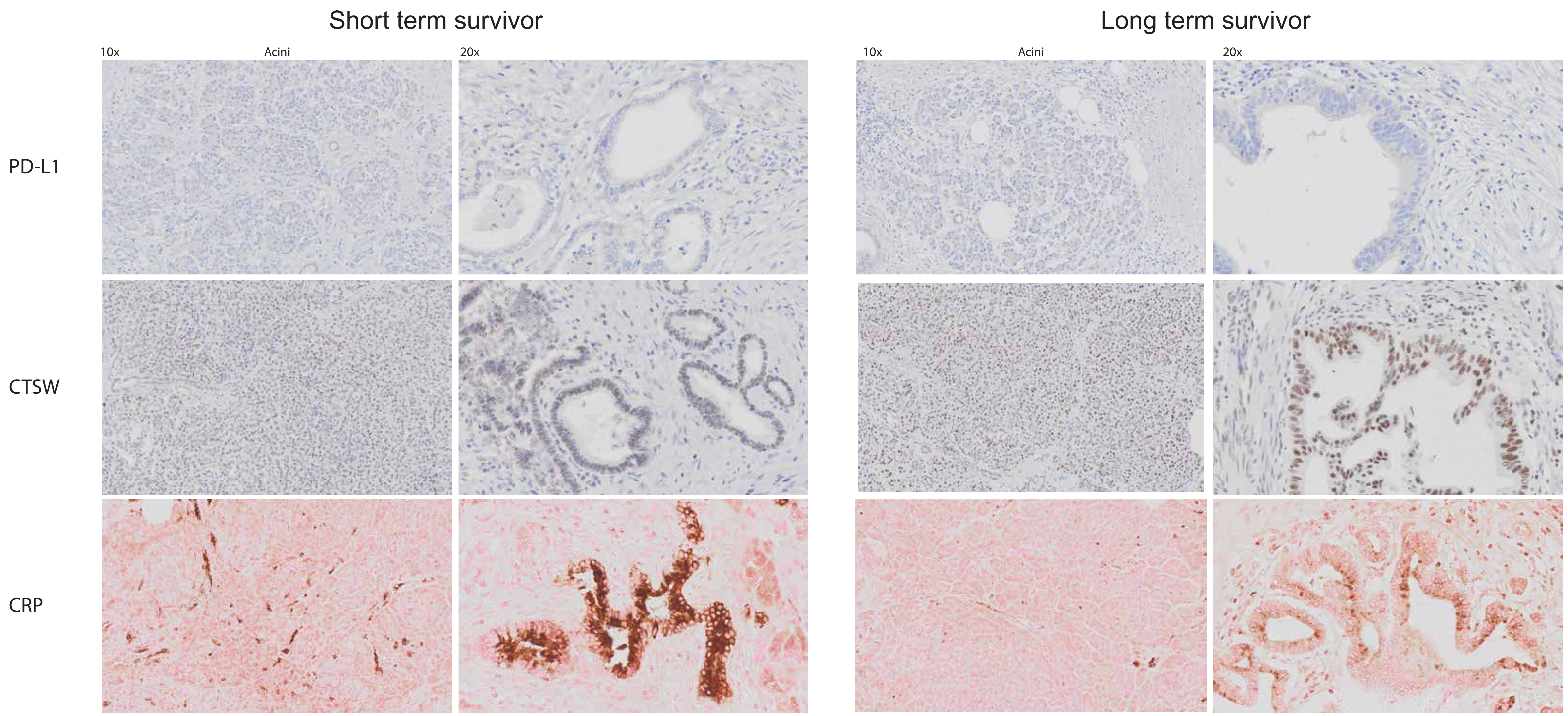

Supplement: Supplementary file 2 — Figure S1 Volcano plots depicting the p value of differentially expressed genes as a function of fold change between the indicated groups. A. OS > 24 months versus OS < 24 months. B. PFS > 12 months versus PFS < 12 months. Colored dots indicate CD274, CTSW, and CRP, principal markers investigated in this study. Figure S2 IHC analysis of PD‐L1, CTSW, and CRP in short term (9.66 months) and long‐term (30.32 months) overall survivors from the GAP trial. IHC staining is shown in the acini and in the tumor. PD‐L1, CTSW, and CRP peroxidase staining show as brown, and nuclei are counterstained with hematoxylin. Original magnification 10× and 20× as indicated. [file AJCO-21-77-s002.pdf]
